# Response of the microbial community structure to the environmental factors during the extreme flood season in Poyang Lake, the largest freshwater lake in China

**DOI:** 10.3389/fmicb.2024.1362968

**Published:** 2024-04-03

**Authors:** Li Zhang, Lijuan Yuan, Jianjun Xiang, Qiegen Liao, Dawen Zhang, Jutao Liu

**Affiliations:** ^1^Institute of Quality & Safety and Standards of Agricultural Products Research, Jiangxi Academy of Agricultural Sciences, Nanchang, Jiangxi, China; ^2^Jiangxi Provincial Institute of Water Sciences, Nanchang, Jiangxi, China

**Keywords:** Poyang Lake, bacterial diversity, extreme flood season, environmental factors, microbial community

## Abstract

**Background:**

Poyang Lake is the largest freshwater lake in China, and there are several studies on the composition and diversity of bacteria in Poyang Lake, while few quantitative studies were carried out on the response of the bacterial community to environmental factors during the extreme flood season in Poyang Lake.

**Methods:**

The connected-lake heterogeneity of bacterial community composition (BCC) was investigated in Poyang Lake during the flood season in 2020. Illumina high-throughput sequencing technology was used in this study.

**Results:**

The bacterial community structure in the water was different from that in the sediment of Poyang Lake during extreme flood seasons. The bacterial diversity in water was much lower than that in sediment. In the water column, the dominant phyla were *Actinobacteriota*, while the composition of bacteria in sediment was more complex than that in water, and the dominant phyla in sediment were *Proteobacteria, Chloroflexi, Acidobacteriota*, and *Actinobacteriota*. The bacterial diversity in the water of Poyang Lake showed seasonal dynamics, while no seasonal variation of bacterial communities in sediment was observed. The bacterial community structure in the sediment from the two bays and channel areas of Poyang Lake can be distinguished from each other. The microbial diversity in sediment gradually increased from the Sancha Bay to the Zhouxi Bay and then to the channel, but the total nitrogen (TN) concentration in sediment (STN) and the total phosphorus (TP) concentration in sediment (STP) showed opposite trends. This might be due to the anthropogenic disturbances from the extreme flood. The bacterial community structure in, water column was significantly correlated with WT, NH4-N, STP, SOM, Chl a, DO, TP, and Eh, while the bacterial community structure in sediment was significantly correlated with SOM and STP.

**Conclusion:**

The bacterial community structure in water was greatly different from that in sediment in Poyang Lake during extreme flood seasons. The bacterial community structure in the water column was not only sensitive to the geochemical characteristics of the water but also affected by some nutrient concentrations in the sediment. During the wet seasons, bacterial diversity was only affected by SOM and STP.

## 1 Introduction

It is well known that bacteria play an important role as the major primary producers in the freshwater lake ecosystem (Newton et al., 2011). The bacterial community characteristics in water and sediment can potentially indicate the health of the freshwater ecosystem due to their basic biochemical cycling position in lake systems (Lindström and Östman, 2011). Therefore, a comprehensive understanding of the bacterial diversity and distribution characteristics in freshwater lake ecosystems is meaningful for better management and the maintenance of the lake’s ecological environment.

Poyang Lake is the largest freshwater lake in China and is the most important wintering site for the East Asian and Australian Flyways. The hydrological conditions in Poyang Lake are quite different than those in disconnected lakes. Poyang Lake has rapidly changing terrain and complex hydrodynamics, which have then formed its unique natural geographical landscape. The water level of Poyang Lake changes drastically throughout the year, with 8.7–22 m. Due to the strong disturbance of natural and human activities, natural and anthropogenic inputs of nutrients and xenobiotics in Poyang Lake have also consistently increased (Ni et al., 2020). The trophic level index (TLI) of Poyang Lake had increased from 46.08 to 56.38 and achieved the light eutropher level (Liu et al., 2023). Therefore, the health threat to the Poyang Lake ecosystem is becoming increasingly serious.

There have been several studies on the composition and diversity of bacteria in Poyang Lake, and many have focused on the relationships between bacterial communities and the physicochemical properties of the lake (Ding et al., 2015; Ren et al., 2019). The bacterial distribution in lakes is affected by many physicochemical factors, including water temperature (WT), oxygen concentration, light intensity, illuminant time, seasonal variation, and the degree and type of water pollution (Adams et al., 2015; Felfödi, 2020; Zhang et al., 2020). Shallow groundwater samples in the Poyang Lake basin showed that the bacterial community structure of high-nitrate groundwater is different from that of low-nitrate groundwater (Dong et al., 2019). The spatial distribution patterns of bacterial community composition (BCC) in the surface sediments from the main basins and mouths of major rivers that discharge into the Poyang Lake varied largely among sampling sites (Kou et al., 2016). The anthropogenic disturbances also have significant impacts on the composition and metabolic function of the bacterial community (Jin et al., 2019). Water level fluctuations had significant impacts on the bacterial communities of Poyang Lake. The bacterial communities are taxonomically sensitive in the dry season while more functionally sensitive in the wet season (Ren et al., 2019). It is widely recognized that water and sediment are both indispensable components of a lake, and the habitats are interactional and interdependent in the aquatic environment. Frequent resuspension and deposition of materials (e.g., bacteria) in the water column and sediment were observed in the lake (Qian et al., 2018). There have been many studies that examined the bacterial communities in the water and sediment of Poyang Lake (Dong et al., 2019; Jin et al., 2019; Sun et al., 2020; Guo et al., 2023). For example, Guo et al. (2023) studied the influence of sediment-to-soil conversion on microbial composition and stability in Poyang Lake in December. Sun et al. (2020) examined the bacterial diversities and community compositions in freshwater and sediment niches and explored the relationship between environmental parameters and the diversity and structure of bacterial communities in Poyang Lake, but this study was focused on the entrance area of five tributaries of Poyang Lake. In addition, there has been no study on the bacterial communities and the response of the bacterial communities to environmental factors when the extreme flood occurred in Poyang Lake. Moreover, the bacterial community from the water and sediment of Poyang Lake and the correlation between the bacterial community and the water and sediment need to be extensively studied (Ding et al., 2015; Kou et al., 2016; Ren et al., 2019; Sun et al., 2020).

However, with global warming, the frequency and intensity of extreme precipitation events have increased. In 2020, rainfall in the Yangtze River basin will reach 1441.5 mm, 22% more than usual and the most since 1961 (Chen, 2020; Huang et al., 2021). Under the influence of heavy rain in the middle and lower reaches of the Yangtze River, Poyang Lake experienced a large-scale flood. The water level when we sampled in July was 21.74 m (measured at Xingzi Station, an iconic hydrological station on Poyang Lake), which was at the historic high level of Poyang Lake (Liao et al., 2021). Thus, the aims of the present study are to (1) analyze the bacterial diversity comprehensively and (2) evaluate the effects of environmental factors on the bacterial community in Poyang Lake ecosystems during the extreme flood season.

## 2 Materials and methods

### 2.1 Study site description and sample collection

**Figure 1:**
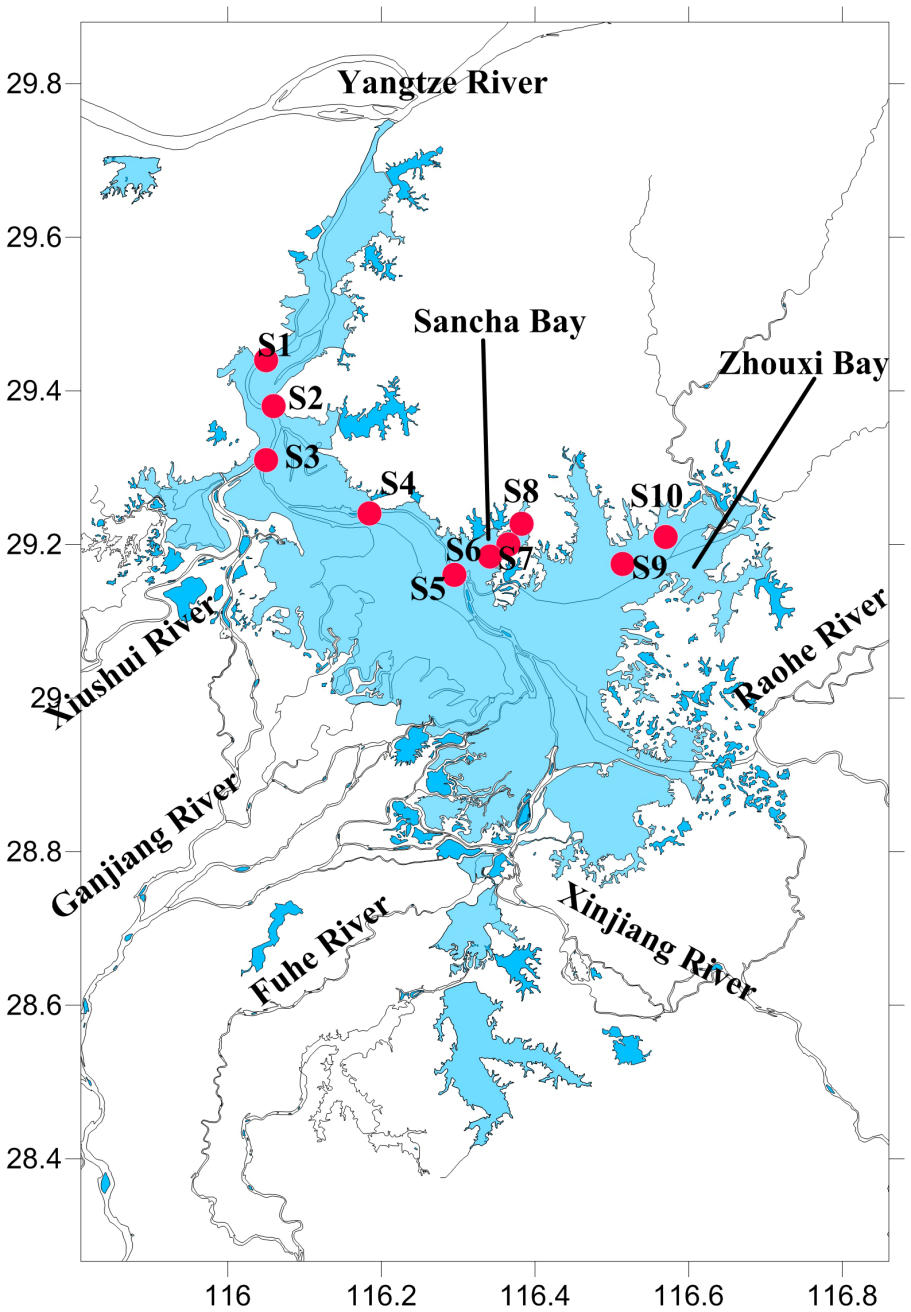
The map of Poyang Lake with sampling stations.

Poyang Lake (28°52′21″–29°06′46″N, 116°10′24″–116°23′50″E) is located in the middle and lower reaches of the Yangtze River, northern Jiangxi Province, China (Figure 1). It is the largest freshwater lake and Yangtze-connected lake in China, with a storage capacity of 2.95 billion m^3^. Each year, approximately 1.457 × 10^11^ m^3^ of water from Poyang Lake flows into the Yangtze River, which accounts for approximately 15.6% of the Yangtze River’s water volume.

The water and sediment samples were collected from June to October 2020 in Poyang Lake, when the water level (measured at Xingzi Station, an iconic hydrological station on Poyang Lake) was from 16.16 to 21.74 m. A total of 10 sample sites were set. Sites 1, 2, 3, 4, and 5 were set in the channel area, where there is always water throughout the year. Sites 6, 7, and 8 were set in the Sancha Bay, which was a closed branch and not connected with other rivers, and the water depth (WD) was too low to get to this area by boat during the dry season. Sites 9 and 10 were located near the Zhouxi Bay, where a branch of the lake was connected to the inflow mouth of a river (Figure 1).

Water samples at each site were collected from surface water (top 50 cm) with a 5-L Schindler sampler. In total, 300 mL of water samples from each site were filtered through a 0.2 μm polycarbonate membrane filter (Whatman, United Kingdom). The filter membranes were immediately frozen in liquid nitrogen and then stored at −80°C in the laboratory until DNA extraction. Sediment samples were collected simultaneously from the same sites (top 5 cm) with an Ekman grabber. A total of 10 g of sediment samples were collected in sterile self-sealed tubes and stored as the same as the filter membranes before. Duplicate water and sediment samples were collected to measure physical and chemical parameters.

### 2.2 Physicochemical parameters of water and sediment

#### 2.2.1 Water

In the field, WT, pH, oxidation–reduction potential (Eh), conductivity (CO), and dissolved oxygen (DO) were determined using a five-star portable multi-parameter measuring instrument (Orion, CO, USA). Transparency (TR) was measured by a Secchi disk, and the WD was measured using a digital ultrasonic echo sounder (Umwelt and Wissenschafts Technik, Mondsee, Austria). Chlorophyll a (Chl *a*) and chlorophyll b (Chl *b*) were analyzed according to the Chinese Water Resources Industry Standard (SL 88–2012) (Department of Water and Soil Conservation, 2012). Chemical oxygen demand (COD_Mn_), total nitrogen (TN), total dissolved nitrogen (DTN), total phosphorus (TP), total dissolved phosphorus (DTP), ammonium (NH_4_), nitrite (NO_2_), and nitrate (NO_3_) contents in the water sample were analyzed according to the American Public Health Association (1998). Phycocyanin (PC) in the water sample was analyzed according to the method of Zhang et al. (2013).

#### 2.2.2 Sediment

Total nitrogen concentration in sediment (STN), total phosphorus concentration in sediment (STP), and organic matter in sediment (SOM) were analyzed according to the method of Jin and Tu (1990).

##### 2.2.2.1 DNA extraction and PCR amplification

DNA extraction and PCR amplification were analyzed according to the methods of He et al. (2017). Genomic DNA was isolated from samples with the PowerWater DNA Isolation Kit (Mo Bio Laboratories, Solana Beach, CA, USA) according to the manufacturer’s instructions. Extracted genomic DNA was detected by 1% agarose gel electrophoresis. An aliquot of 10 ng purified DNA template from each sample was amplified in a 50 μL reaction system with the following conditions: 1 cycle of 95°C for 3 min followed by 30 cycles of denaturation at 95°C for 30 s, annealing at 55°C for 30 s, extension at 72°C for 45 s, and a final extension at 72°C for 10 min. The primer pairs 338F (5′-ACTCCTACGGGAGGCAGCAG-3′) and 806R (5′-GGACTACHVGGGTWTCTAAT-3′) were used for the 16S rRNA amplification (Xu et al., 2016). PCR was carried out on a GeneAmp 9700 PCR system (Applied Biosystems, Foster City, CA, USA). The PCR-amplified products were visualized on agarose gels (2% gel electrophoresis) and purified with a DNA gel extraction kit (Axygen Biosciences, Union City, CA, USA). PCR products were quantified using the QuantiFluor^™^-ST fluorometer quantitative system (Promega Biotech, Beijing, China) and mixed with the appropriate proportion based on sequencing requirements. Sequencing was conducted by Majorbio Bio-Pharm Technology Co., Ltd., Shanghai, China, using an Illumina MiSeq platform (Illumina, San Diego, CA, USA).

### 2.3 Processing of Illumina sequencing data

Before analysis, raw FASTQ files were demultiplexed and quality-filtered using the software package Quantitative Insight into Microbial Ecology (QIIME, version 1.9.1). The overlapped paired-end sequences were assembled with FLASH. Bacterial reads were truncated at any site receiving an average quality score of <20 over a 50 bp sliding window, discarding the truncated reads that were shorter than 50 bp. Any read containing one or more ambiguous base calls (“N”) was discarded. In addition, the truncated reads of <80% (of the raw read length) of consecutive high-quality base calls were discarded. Only sequences that overlapped longer than 10 bp were assembled according to their overlap sequence. Unique sequences were clustered into operational taxonomic units (OTUs), defined as having at least 97% similarity. Taxonomic assignment on the OTUs was performed using the ribosomal database project (RDP) classifier (version 2.2)^1^ against the SILVA (Release132)^2^ ribosomal RNA gene database with a minimum confidence threshold of 0.7. Sequences representing chloroplasts and mitochondria were filtered out. Shared and unique OTUs were graphically represented in Venn diagrams as described in Chen and Boutros (2011).

### 2.4 Statistical analyses

Sobs richness estimates, Shannon’s diversity index values, Shannon’s evenness index values, and the Good’s coverage were calculated in MOTHUR for total, and the evaluation index is used in the OTU at a similar level of 97% (0.97). The unweighted pair-group method with arithmetic mean (UPGMA) was selected for beta diversity to capture phylogenetic distance while not taking relative abundance into consideration. UniFrac distance metrics analysis was performed using OTUs for each sample, and principal coordinate analysis (PCoA) and hierarchical clustering analysis were computed using QIIME software (Version 1.9.1)^3^ with unweighted_uniFrac distance. PCoA was conducted based on RDP classifier results from MOTHUR, OTUs, and weighted UniFrac. Linear discriminant analysis (LDA) effect size (LEfSe) was used to elucidate the differences among bacterial taxa. The LDA score of ≥4 was considered to be an important contributor to the model. The relationship between environmental variables and the bacterial communities in Poyang Lake was analyzed by redundancy analysis (RDA)/canonical correlation analysis (CCA). Redundant variables were eliminated by functions of envfit (permu = 999) and vif.cca. The comparison of predicted functional profiles between water and sediment habitats was examined by using FAPROTAX based on raw OTU tables. It was defined as significant when the Welch *t*-test yielded a value of *p* of <0.05. The shifts in the relative abundance of bacterial phyla were displayed by a heat map, which was modeled with a vegan package in R. The Spearman correlation coefficients of the top 20 abundant bacterial phyla and environmental factors were calculated and displayed on the heat map.

## 3 Results

### 3.1 Physicochemical variables

Significant fluctuations in the physicochemical properties were observed in each sample site from June to October 2020, while there were obvious differences between different sampling sites during the same time as well. The results of physicochemical parameters in water and sediment samples are shown in Table 1. In the water column, the values of TR, pH, Eh, WT, CO, DO, suspended substance (SS), TN, DTN, TP, DTP, NO_3_-N, NO_2_-N, NH_4_-N, PO_4_-P, COD_Mn_, Chl *a*, Chl *b*, and PC ranged from 50 to 150 cm, 7.38 to 8.80, 134.90 to 437.30 mV, 19.08 to 34.10°C, 0.07 to 0.14 ms/cm, 3.62 to 12.88 mg/L, 0.80 to 38.50 mg/L, 0.92 to 2.84 mg/L, 0.80 to 1.87 mg/L, 0.03 to 0.19 mg/L, 0.01 to 0.11 mg/L, 0.19 to 1.10 mg/L, 0.00 to 0.07 mg/L, 0.15 to 0.62 mg/L, 1.00 to 21.00 μg/L, 2.12 to 4.57 mg/L, 3.64 to 61.75 μg/L, 0.02 to 50.91 μg/L, and 14.29 to 489.60 μg/L, respectively, and with the average values of 93.76 cm, 8.02, 252.65 mV, 26.71°C, 0.10 ms/cm, 7.83 mg/L, 9.84 mg/L, 1.42 mg/L, 1.18 mg/L, 0.08 mg/L, 0.05 mg/L,0.53 mg/L, 0.01 mg/L,0.32 mg/L, 7.10 μg/L, 2.99 mg/L, 34.53 μg/L, 18.28 μg/L, and 64.22 μg/L, respectively. In sediment, the contents of STN, STP, and SOM ranged from 1.49 to 11.56 mg/kg, 0.13 to 1.41 mg/kg, and 2.44 to 10.76 g/100 g, respectively, with mean values of 5.01 mg/kg, 0.78 mg/kg, and 6.04 g/100 g, respectively.

**Table 1:** Physicochemical parameters in water and sediment samples collected from Poyang Lake.

Parameters	Mean	Maximum	Minimum
Transparency (cm)	93.76	150.00	50.00
pH	8.02	8.80	7.38
Eh (mV)	252.65	437.30	134.90
Water temperature (°C)	26.71	34.10	19.08
Conductivity (ms/cm)	0.10	0.14	0.07
DO (mg/L)	7.83	12.88	3.62
SS(mg/L)	9.84	38.50	0.80
TN(mg/L)	1.42	2.84	0.92
DTN(mg/L)	1.18	1.87	0.80
TP(mg/L)	0.08	0.19	0.03
DTP(mg/L)	0.05	0.11	0.01
NO_3_-N(mg/L)	0.53	1.10	0.19
NO_2_-N(mg/L)	0.01	0.07	0.00
NH_4_-N(mg/L)	0.32	0.62	0.15
PO_4_-P(μg/L)	7.10	21.00	1.00
COD_Mn_(mg/L)	2.99	4.57	2.12
Chl *a*(μg/L)	34.53	61.75	3.64
Chl *b*(μg/L)	18.28	50.91	0.02
PC (μg/L)	64.22	489.60	14.29
STN (mg/kg)	5.01	11.56	1.49
STP(mg/kg)	0.78	1.41	0.13
SOM(g/100 g)	6.04	10.76	2.44

### 3.2 Diversity of bacterial communities in water and sediment samples

**Figure 2:**
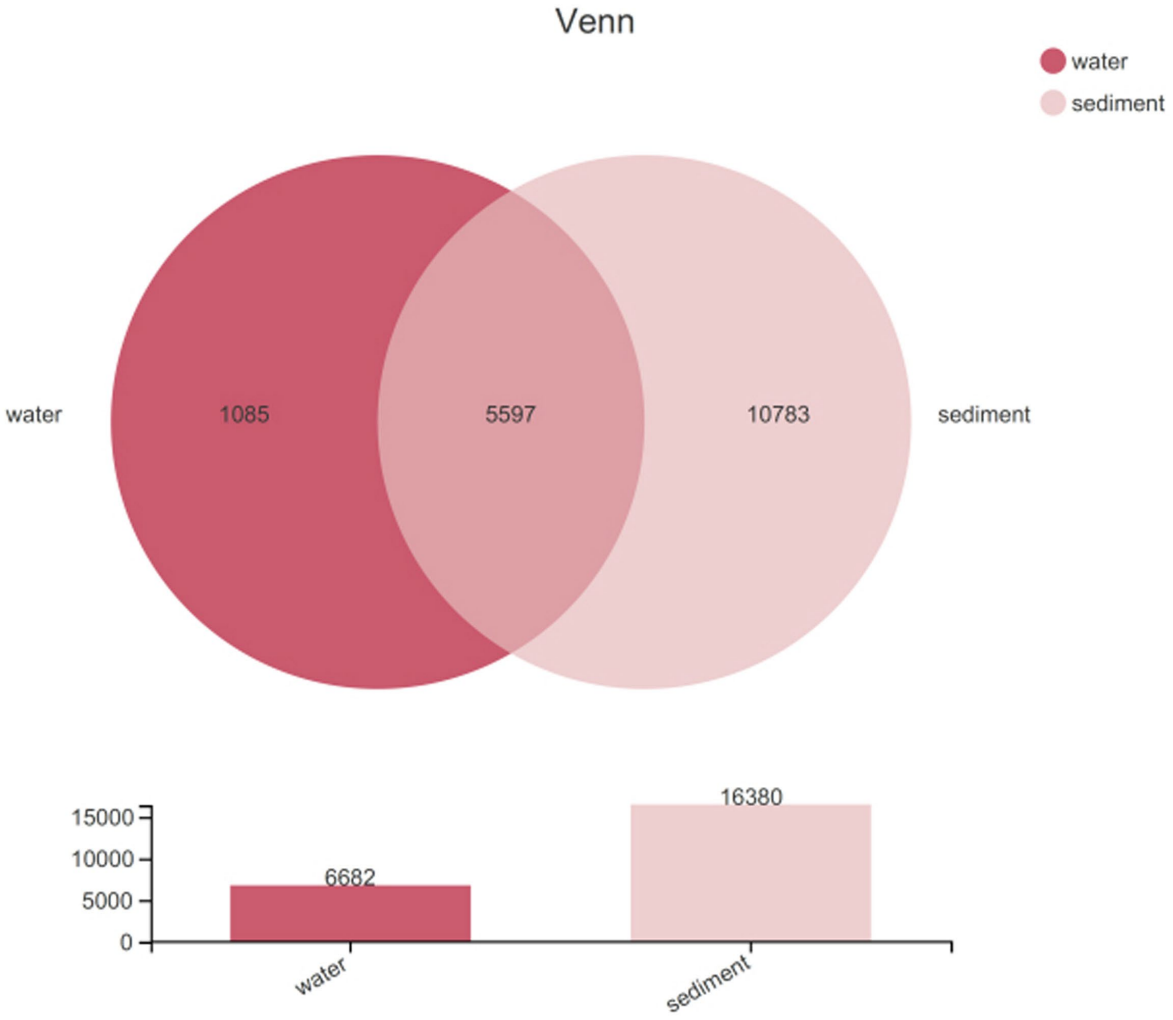
A Venn diagram showing the unique and shared OTUs in water column and sediment.

Sequencing data were successfully generated from 34 water samples and 49 sediment samples. DNA from all water samples in July was not successfully extracted. A total of 3,810,287 16S rRNA sequence tags were generated through pyrosequencing, with an average read length of approximately 416 bp that ranged in size from 202 to 534 bp and clustered into 17,465 OTUs at a 97% nucleotide similarity level. The microbial diversity in sediment (16,380 OTUs) was higher than that in water (6,682 OTUs) samples; meanwhile, unique species accounted for 16.24% of all 6,682 OTUs in water and 65.83% of all 16,380 OTUs in sediment, and the number of shared OTUs between water and sediment was 5,597 (Figure 2). All data were normalized to the smallest sequence tag, and then the data were reanalyzed to show a normal distribution of variances (Table 2).

**Table 2:** Normalized summary of sequence library, OTUs, and bacterial diversity indices of water column samples and sediment samples from Poyang Lake.

		Nseqs	OTUs	Chao	Shannon	Simpson	Coverage
Sediment	Mean	47941.24	3929.88	5685.09	6.77	0.0058	0.950
	Maximum	84887.00	6045.00	7660.80	7.46	0.0409	0.989
	Minimum	34303.00	1794.00	2207.11	4.80	0.0018	0.904
Water	Mean	42975.47	1043.53	1276.93	4.28	0.0518	0.992
	Maximum	56,596	2,146	2282.87	5.18	0.103	0.999
	Minimum	33,714	471	503.36	3.42	0.0224	0.983

**Figure 3:**
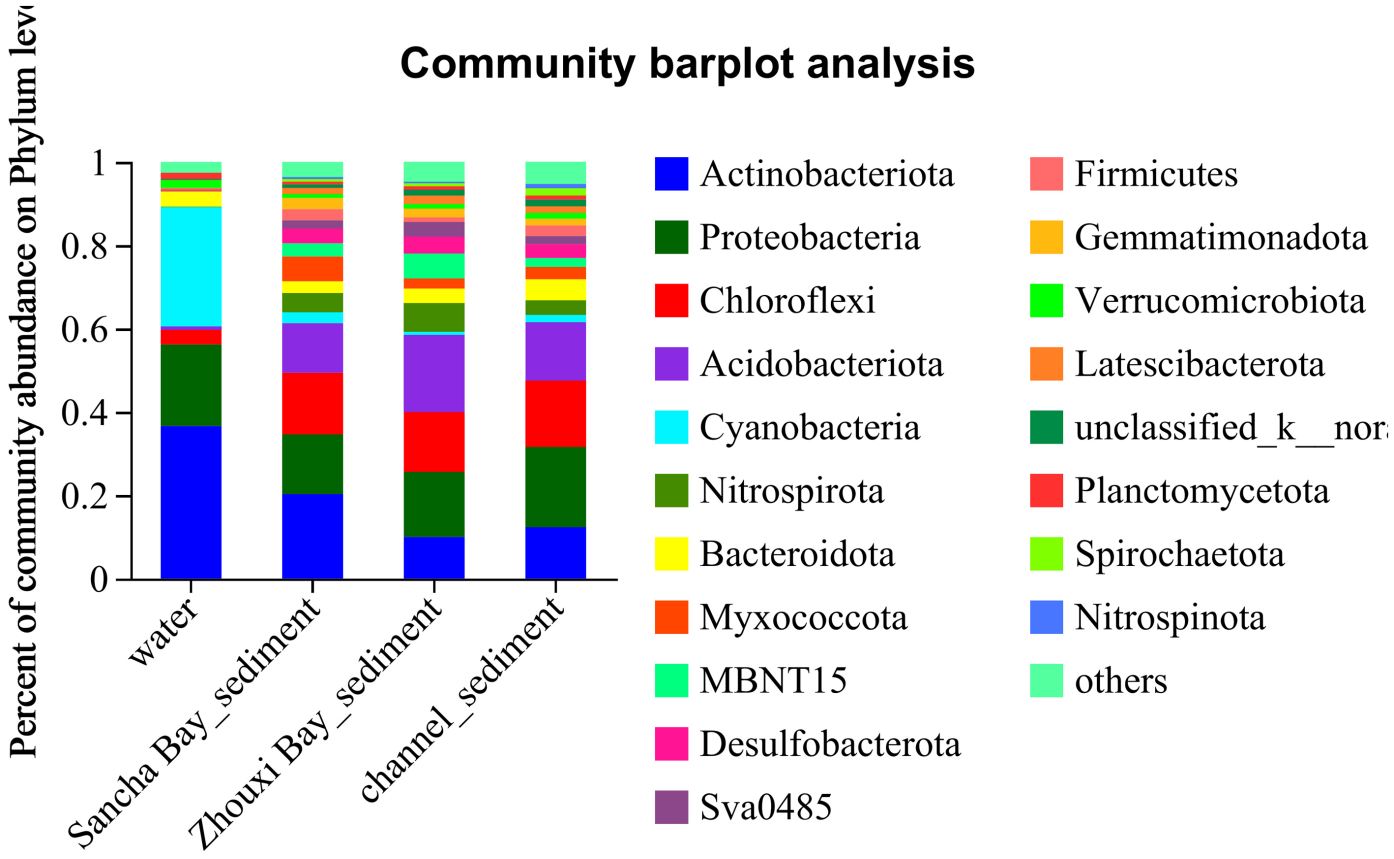
The distribution of microbial community for water and sediment from the Sancha Bay, the Zhouxi Bay, and channel areas on the phylum level.

In the water column, the dominant phyla (relative abundance>2%) were *Actinobacteriota* (33.45%), followed by *Cyanobacteria* (29.73%), *Proteobacteria* (22.01%), *Bacteroidota* (4.02%), and *Chloroflexi* (3.06%), making up 91.91% of the total OTUs (Figure 3). During the survey period, *Actinobacteriota* peaked in September, while *Cyanobacteria* peaked in June, and *Proteobacteria* changed indistinguishably. The composition of bacteria in sediment was more complex than in water. *Proteobacteria* (17.63%), *Actinobacteriota* (14.73%), *Chloroflexi* (14.63%), and *Acidobacteriota* (14.49%) occupied the first concentration gradient in sediment, with a total content of 61.48%. *Nitrospirota* (4.59%), *Bacteroidota* (4.04%), *Myxococcota* (3.67%), *Desulfobacterota* (3.49%), *MBNT15* (3.22%), *Gemmatimonadota* (2.50%), *Sva0485* (2.20%), and *Firmicutes* (2.11%) were close behind, with a total content of 25.82% (Figure 3).

**Figure 4:**
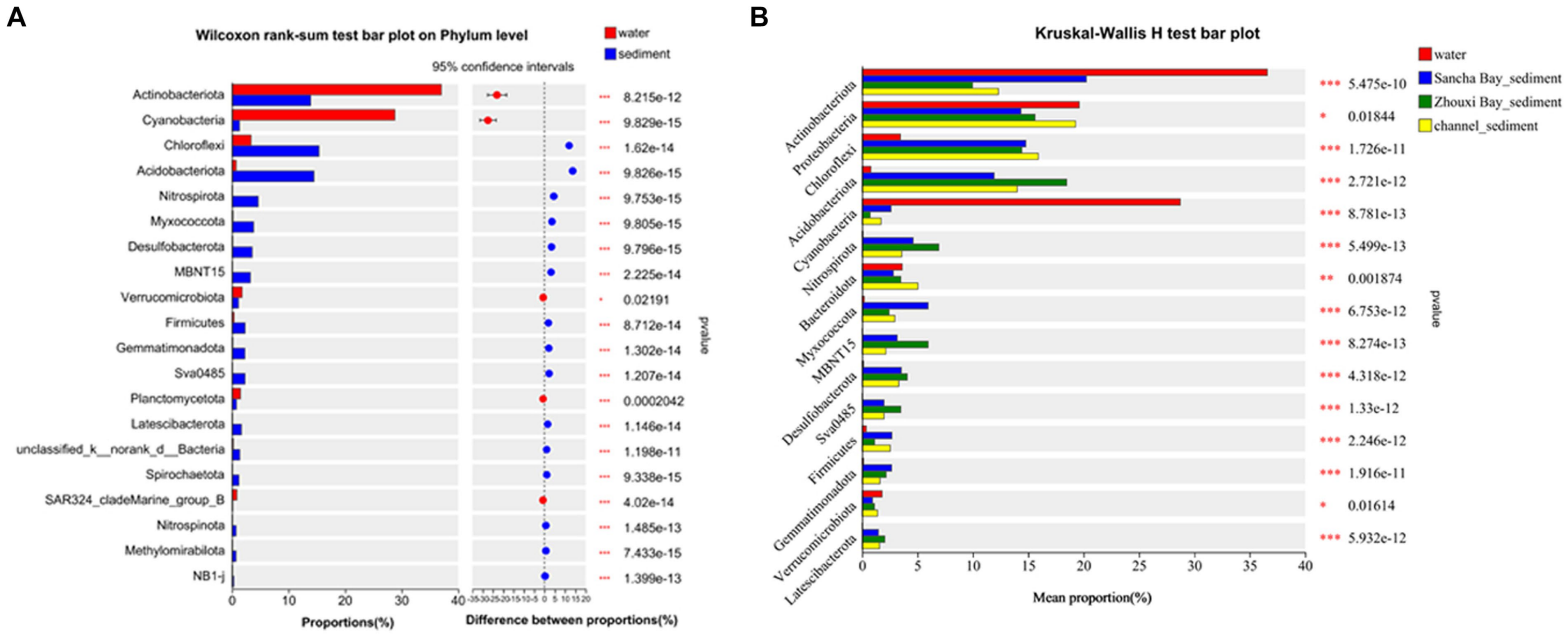
Wilcoxon rank-sum test bar plot on phylum level showing the significant differences between water and sediment **(A)**, Kruskal–Wallis H-test bar plot at the phylum level showing the significant differences among water and sediment from the Sancha Bay, the Zhouxi Bay, and channel **(B)**. Positive differences in mean relative abundance indicate the bacterium is overrepresented on sediment **(A)**, while negative differences indicate greater abundance in water **(A)**. *p < 0.05; **p < 0.01; ***p < 0.001.

Comparing relative abundances of these dominant phyla between the water column and sediment, *Actinobacteriota* (33.45% vs. 14.73%, *p* < 0.001) and *Cyanobacteria* (29.73% vs. 1.48%, *p* < 0.001) had a significantly higher relative abundance in water than in sediment, while *Chloroflexi* (3.06% vs. 14.63%, *p* < 0.001), *Acidobacteriota* (0.72% vs. 14.49%, *p* < 0.001), *Nitrospirota* (0.078% vs. 4.59%, *p* < 0.001), *Myxococcota* (0.19% vs. 3.67%, *p* < 0.001), *Desulfobacterota* (0.14% vs. 3.49%, *p* < 0.001), *MBNT15* (0.032% vs. 3.22%, *p* < 0.001), *Firmicutes* (0.75% vs. 2.11%, *p* < 0.001), *Gemmatimonadota* (0.28% vs. 2.50%, *p* < 0.001), and *Sva 0485* (0.03% vs. 2.20%, *p* < 0.001) had a significantly lower relative abundance in water samples (Wilcoxon rank-sum test) (Figure 4).

**Figure 5:**
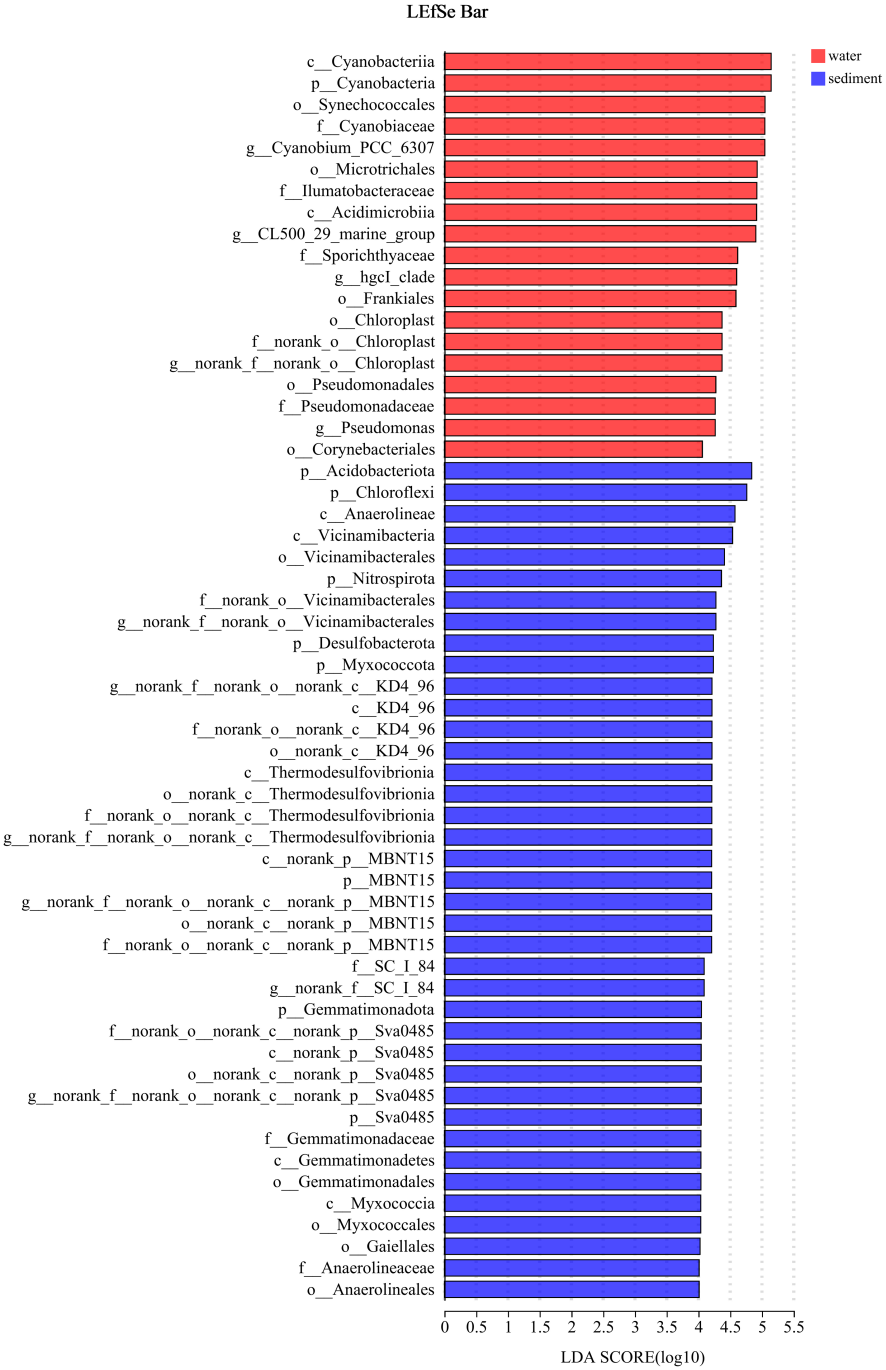
Indicator bacteria with LDA scores of 4 or greater in bacterial communities (phylogenetic levels from phylum to genus associated with water and sediment). Different colored regions represent different constituents (red, water; blue, sediment).

**Figure 6:**
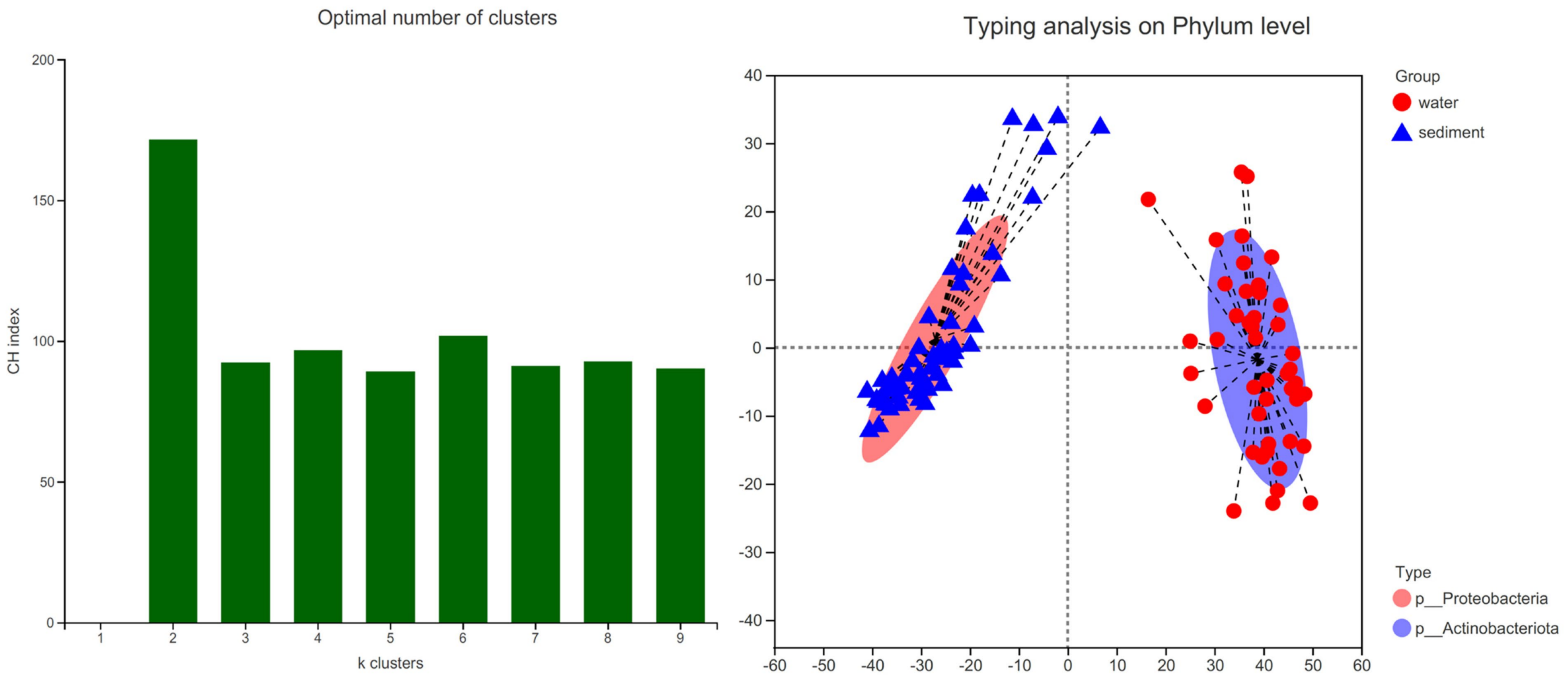
Bacterial type analysis for water and sediment using Jensen–Shannon distance. The left panel shows that the data are most naturally separated into two clusters by the PAM method. The *x*-axis shows the cluster number; the *y*-axis shows the CH index, a measure of cluster separation. The right panel shows the clustering of the first two principal components. The data indicates that water column bacteria belong to the Proteobacteria type, while sediment bacteria belong to the Actinobacteriota type.

The LEfSe method identified a suite of specialized bacterial taxa enriched in the water column and sediment, respectively (Figure 5). Based on the bacteria abundance at the phylum level, partitioning around medoids (PAM) analysis was performed to cluster the microflora at the phylum level (Figure 5, Jensen–Shannon distance). The result showed that the water column and sediment have their own unique bacterial types (Figure 6). The water samples belonged to the *Proteobacteria* type, while the sediment samples belonged to the *Actinobacteriota* type.

**Figure 7:**
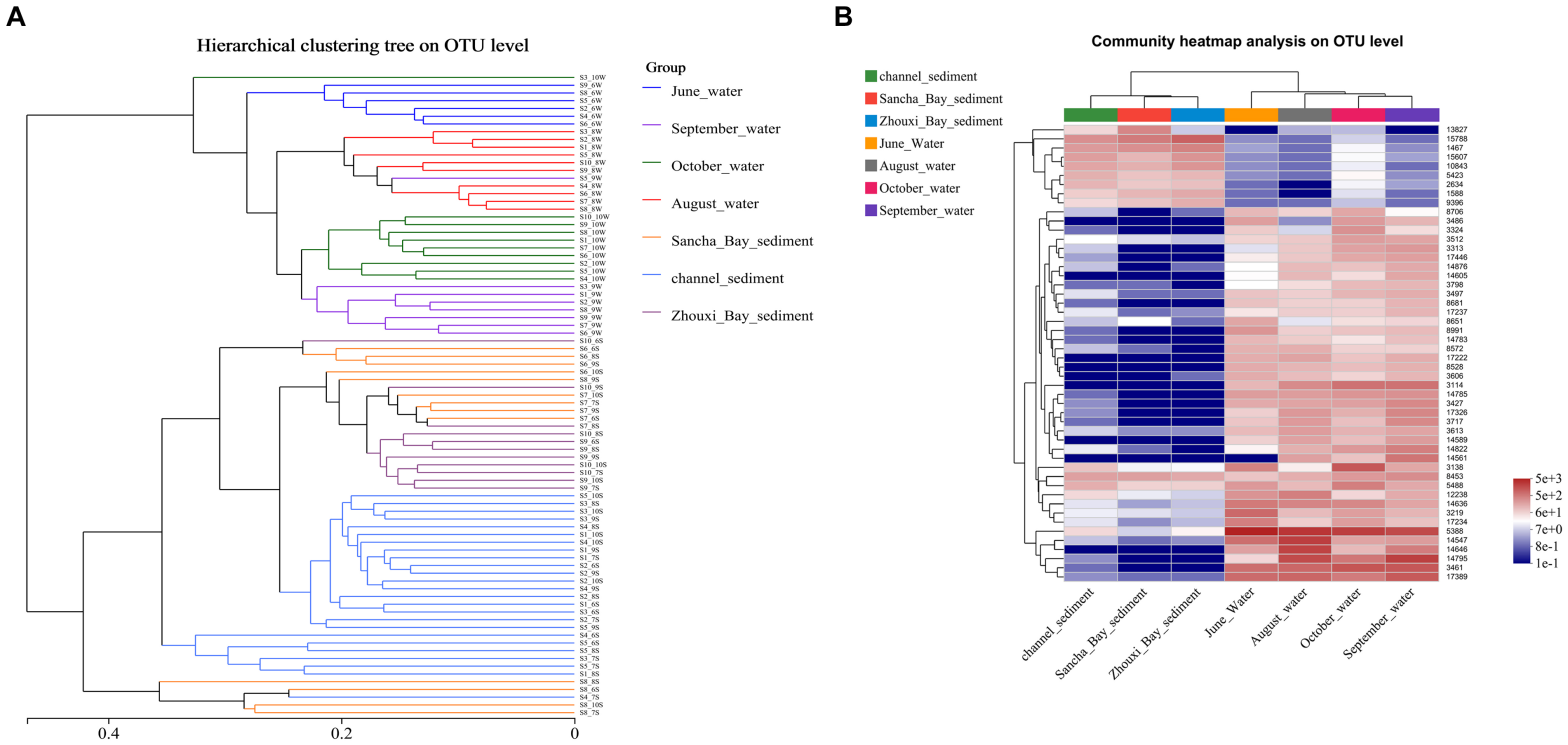
Comparison of water and sediment bacterial communities. **(A)** Hierarchical cluster analysis results based on Bray–Curtis at the OTU level. Unweighted pair-group method with arithmetic mean (UPGMA) analysis of bacterial community structure based on the 16S rRNA gene amplicon sequencing data. **(B)** The heat map showed the relative abundance of the bacterial OTU in water and sediment samples.

To assess changes in the diversity of bacterial communities with each sample, hierarchical cluster analysis was performed (Figure 7A). The resulting dendrograms revealed apparent differences between water and sediment. The samples from water niches clustered closely together, while the samples from sediment formed the other group. Water samples from the same month were clustered closely together. On the whole, sediment samples from 10 sites were separated into three groups: the channel areas group (namely sites 1–5), the Lake Bay group (sites 6–8), and the Zhouxi Bay group (sites 9 and 10). The same group clustering results were also shown in the community heat map analysis (Figure 7B).

**Figure 8:**
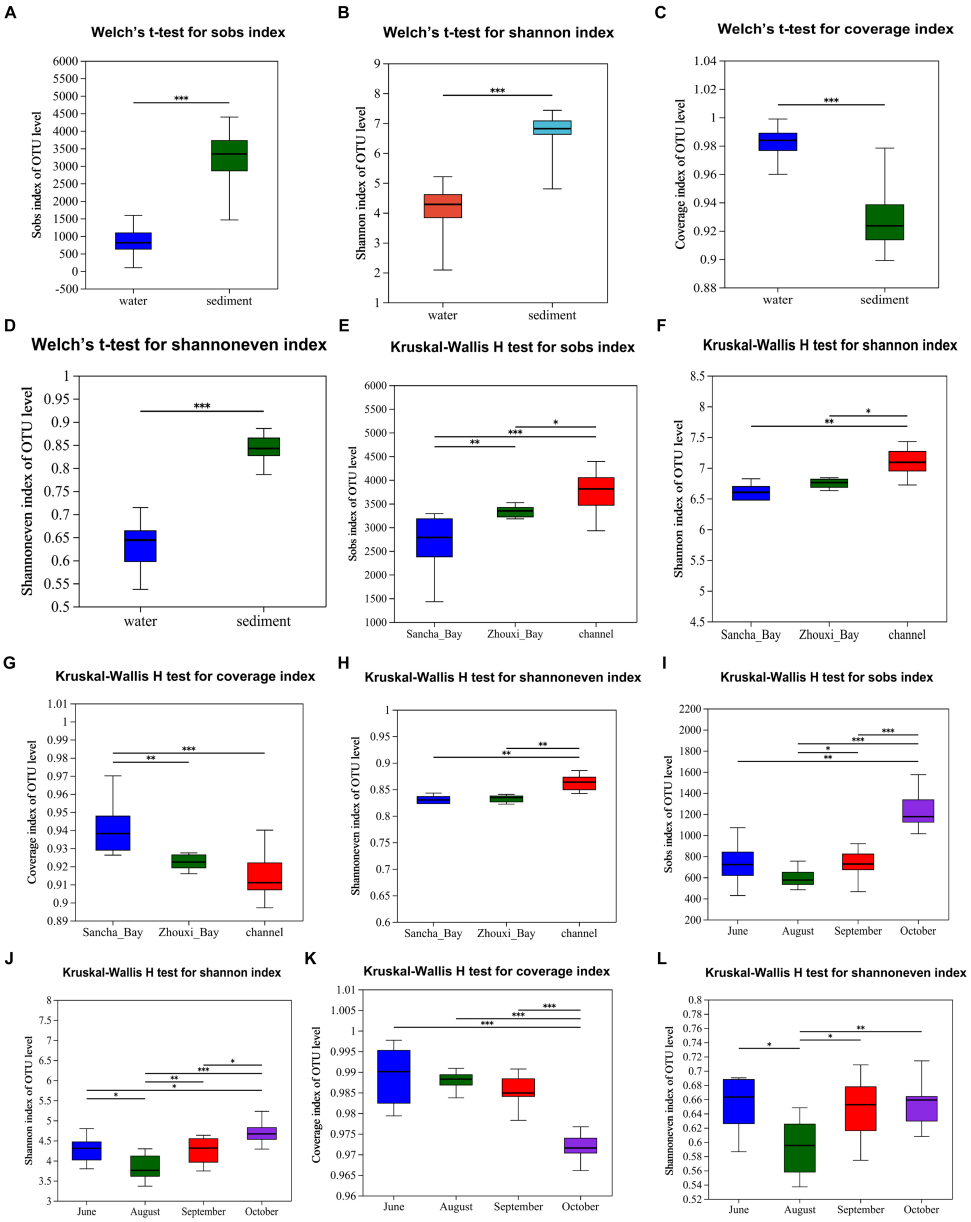
Alpha diversity (Sobs, Shannon, Coverage, and Shannoneven) of bacterial communities in the water column and sediment (**A–D**, Welch’s t-test), Lake Bay sediment, channel sediment (**E–H**, Kruskal–Wallis H-test), and water from different seasons **(I–L)**. *p < 0.05, **p < 0.01, ***p < 0.0001.

The bacterial α-diversity indices of Poyang Lake were much lower in water than that in the sediment (Table 2). Welch’s *t*-test showed an extremely significant difference in alpha diversity between water and sediment samples (*p* < 0.001) (Figures 8A–D). There were significant differences in OTU richness (Sobs) among the sediment from the Sancha Bay, the Zhouxi Bay, and the channel sites. However, no significant difference in the Shannon index was found between the sediment from the Sancha Bay and the Zhouxi Bay. The difference in the Shannon index between the Sancha Bay and the channel area was greater than that between the Zhouxi Bay and the channel area (Figures 8E–H). There were significant differences in the Shannon index among the water from different months except for June and September (Figures 8I–L).

**Figure 9:**
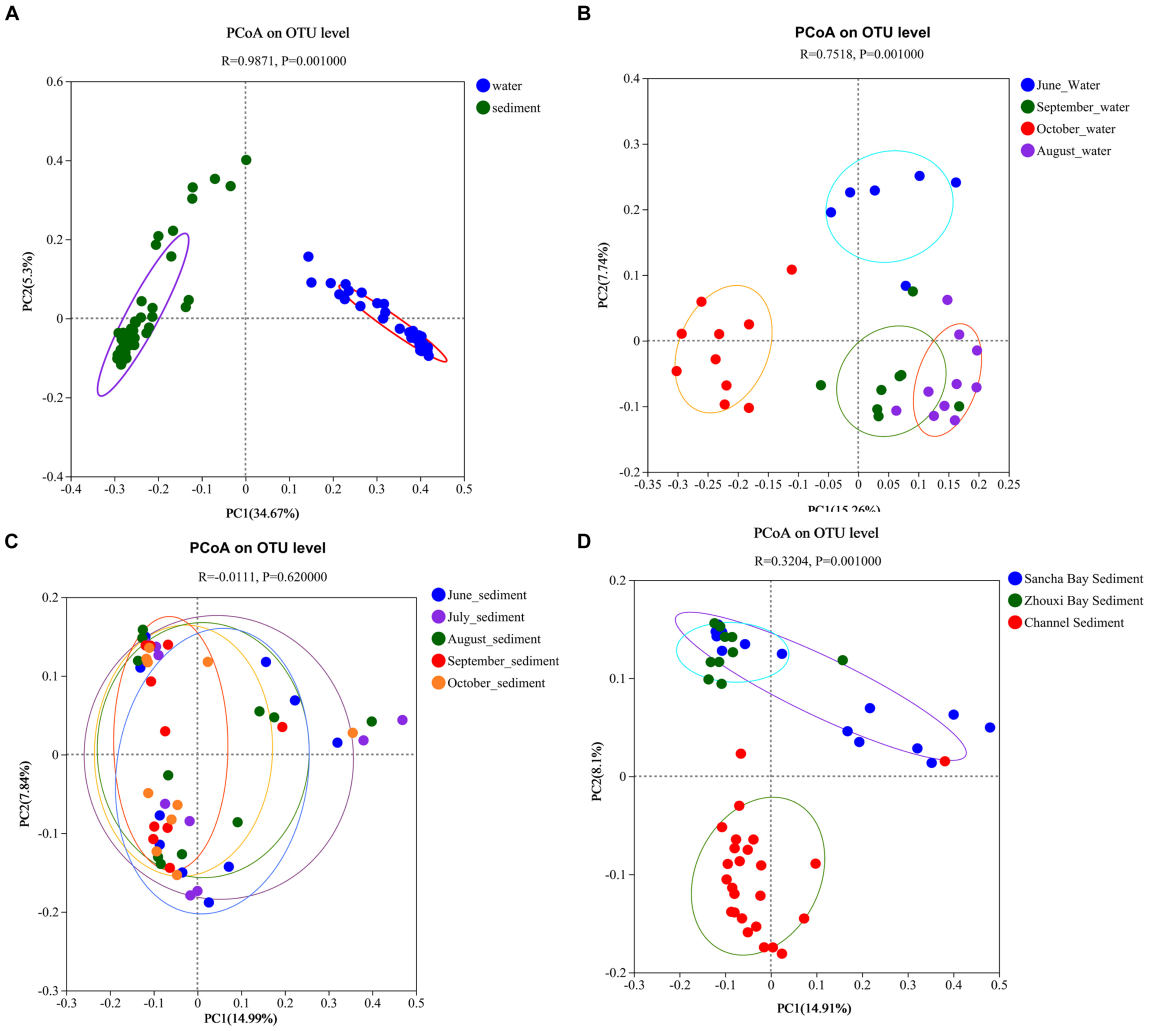
Principal coordinate analysis based on unweighted UniFrac distances between water and sediment **(A)**, among water from different months **(B)**, among sediment from different months **(C)**, and among sediment from different areas **(D)** of Poyang Lake.

Principal coordinates analysis results revealed that the significant differences in bacterial communities between the water column and the sediment were with the first two principal component scores, which accounted for 34.67 and 5.30% of the total variations (*R* = 0.9871, *p* = 0.001) (Figure 9A). The water samples from different months also had obvious different bacterial communities with the first two principal component scores, which accounted for 7.74 and 15.26% of the total variations (*R* = 0.7518, *p* = 0.001) (Figure 9B), while no seasonal variation of bacterial communities in sediment was observed (Figure 9C). Obvious differences in bacterial communities in sediment among two lake bays and channel areas were found [with the first two principal component scores accounting for 8.10 and 14.91% of the total variations (*R* = 0.3204, *p* = 0.001)] (Figure 9D).

### 3.3 Putative function profiles of microbial communities

**Figure 10:**
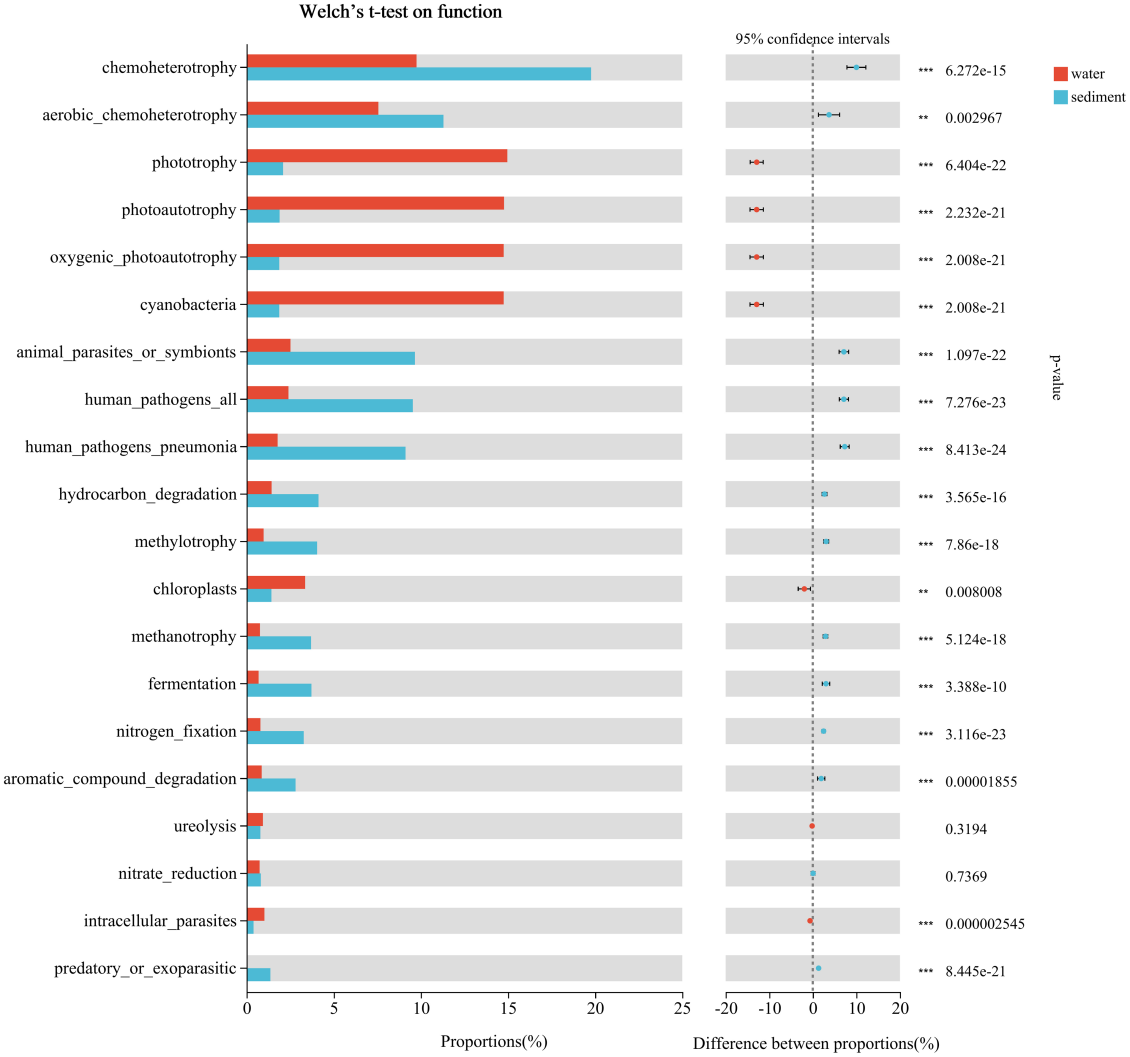
Putative function profiles of microbial communities in the water column and sediment from Poyang Lake.

The predicted functional groups were revealed using FAPROTAX, and a total of 74 functional groups were obtained in Poyang Lake. Among them, chemoheterotrophy, aerobic chemoheterotrophy, phototrophy, photoautotrophy, cyanobacteria, oxygenic photoautotrophy, animal parasites or symbionts, human pathogens pneumonia, hydrocarbon degradation, methylotrophy, chloroplasts, methanotrophy, fermentation, nitrogen fixation, and aromatic compound degradation were the most abundant groups in both habitats (Figure 10). By using Welch *t*-test, the functional groups of chemoheterotrophy (sediment 19.77% vs. water 9.75%, *p* < 0.001), aerobic chemoheterotrophy (11.30% vs. 7.56%, *p* < 0.001), animal parasites or symbionts (9.66% vs. 2.52%, *p* < 0.001), human pathogens all (9.54% vs. 2.41%, *p* < 0.001), human pathogens pneumonia (9.12% vs. 1.78%, *p* < 0.001), hydrocarbon degradation (4.12% vs. 1.43%, *p* < 0.001), methylotrophy (4.05% vs. 0.98%, *p* < 0.001), methanotrophy (3.70% vs. 0.77%, *p* < 0.001), fermentation (3.72% vs. 0.69%, *p* < 0.001), nitrogen fixation (3.28% vs. 0.79%, *p* < 0.001), and aromatic compound degradation (2.81% vs. 0.87%, *p* < 0.001) were significantly enriched in sediment, while the mean proportions of phototrophy (water 14.96% vs. sediment 2.09%, *p* < 0.001), photoautotrophy (14.77% vs. 1.90%, *p* < 0.001), cyanobacteria (14.75% vs. 1.87%, *p* < 0.001), oxygenic photoautotrophy (14.75% vs. 1.87%, *p* < 0.001), and chloroplasts (3.36% vs. 1.42%, *p* < 0.001) were significantly higher in water (Figure 10).

### 3.4 Correlation between bacterial community structure and environmental parameters

**Figure 11:**
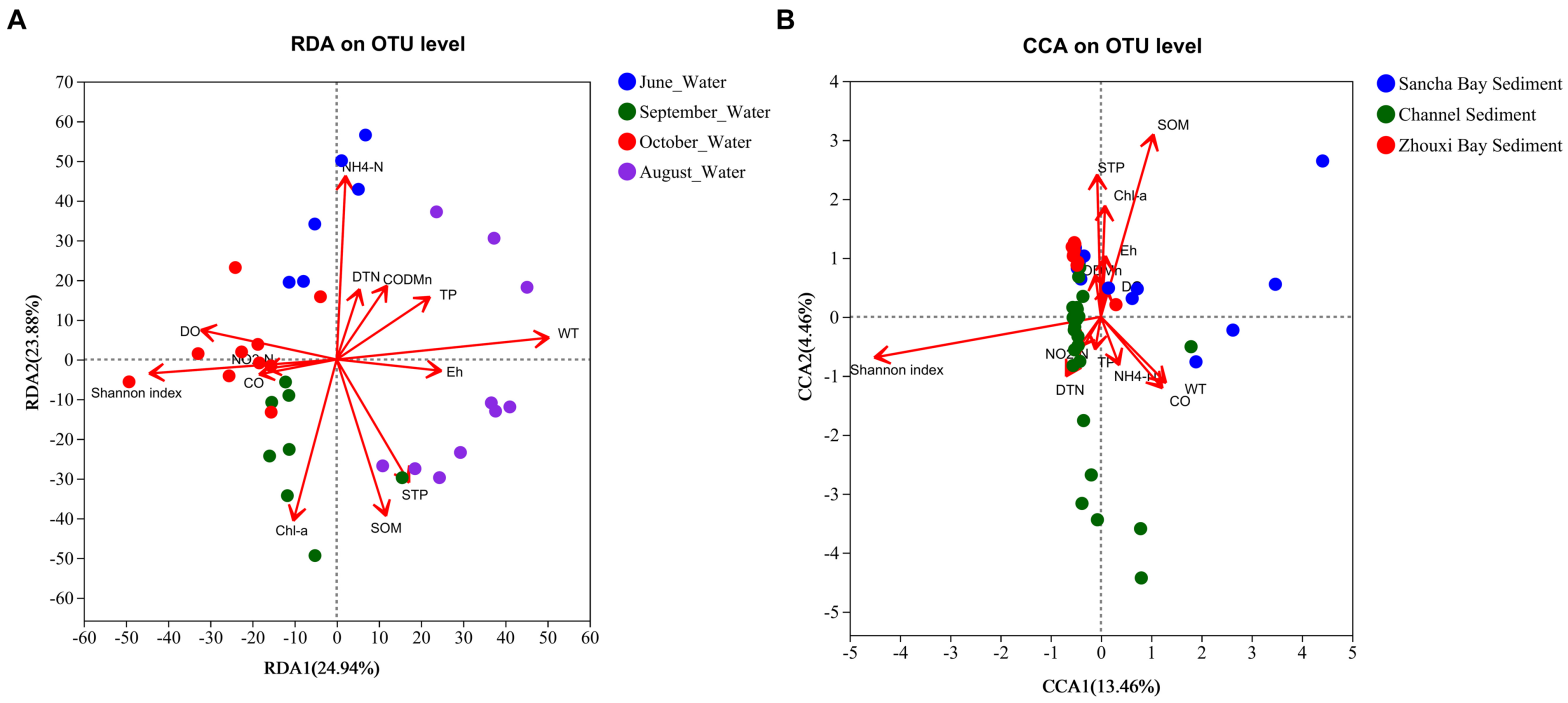
CCA/RDA analysis to show correlations between the bacterial communities and environmental factors in water **(A)** and sediment **(B)** samples from Poyang Lake.

Environmental factors were selected by the functions envfit (permu = 999) and vif.cca, and environmental factors with a value of *p* of >0.05 or vif>10 were removed from the following analysis. The vif values of NO_3_-N, Chl *b*, and STN were higher than 10 and then were removed. An RDA/CCA was conducted to reveal the effect of environmental factors on the microbial community structures in water and sediment. After the removal of the redundant variables, 12 environmental factors were chosen for RDA/CCA, including WT, Eh, CO, DO, DTN, TP, NO_2_-N, NH_4_-N, Chl *a*, COD_Mn_, SOM, and STP. In all the examined environmental factors, WT (*p* = 0.001), NH_4_-N (*p* = 0.001), STP (*p* = 0.002), SOM (*p* = 0.001), Chl *a* (*p* = 0.002), DO (*p* = 0.003), TP (*p* = 0.029), and Eh (*p* = 0.04) were significantly correlated with the water microbial community structure in water (Figure 11A), while SOM (*p* = 0.001) and STP (*p* = 0.041) were important factors that significantly correlated with the microbial community structures in sediment (Figure 11B).

**Figure 12:**
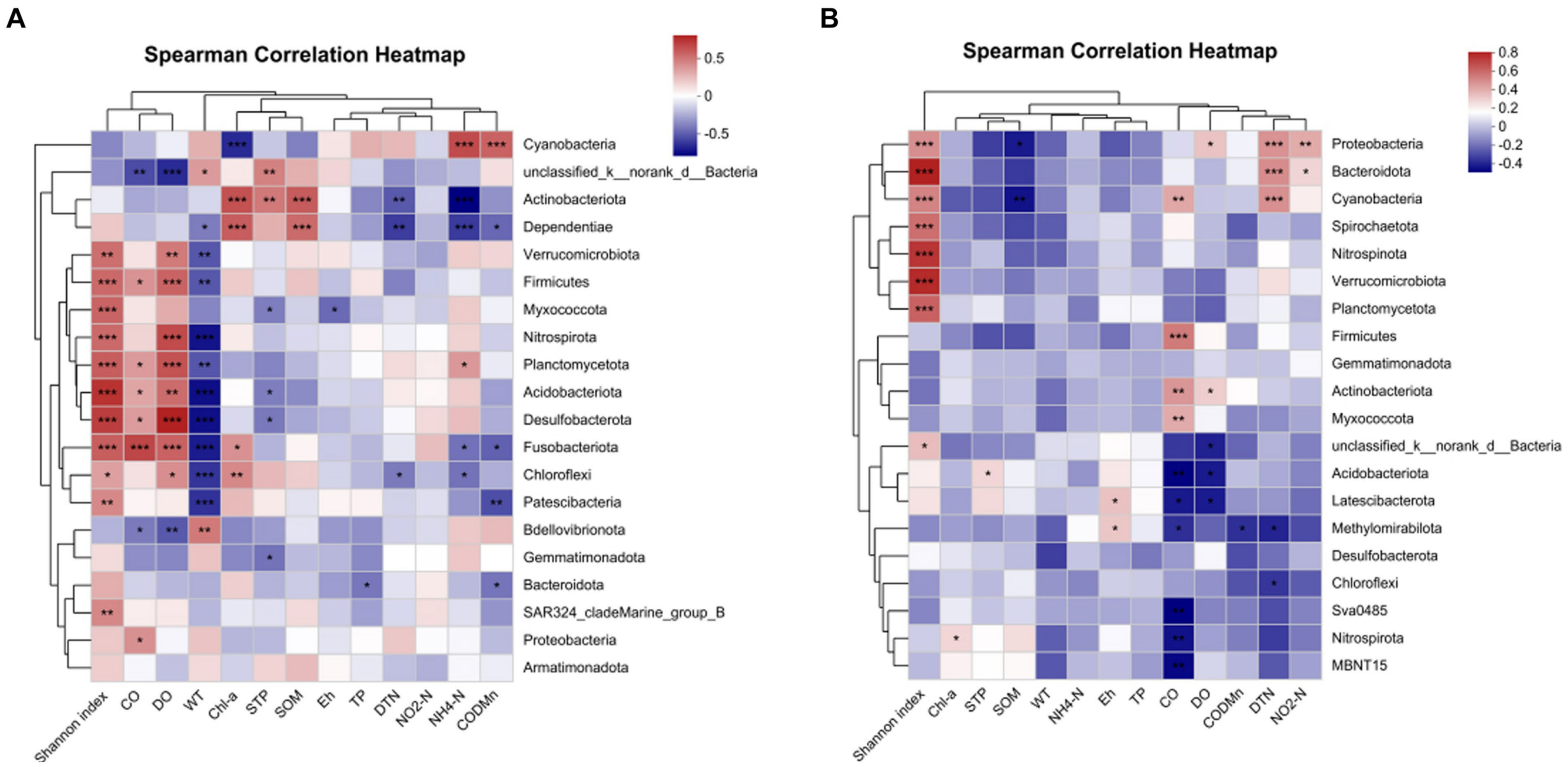
Correlation heat map of the top 20 phyla in water **(A)** sediment **(B)** and environmental factors. *X* and *Y* axes are environmental factors and phyla. R in different colors to show the right side of the legend is the color range of different *R* values. The value of 0.01 < Pnd environmental factors. X and <Pnd environmental factors. *X* and *Y* axes are environmental factors. *P < 0.05, **P < 0.01, ***P < 0.0001.

For more details, the relationship between bacterial phyla and environmental factors was demonstrated by a correlation heat map (Figure 11). In water samples (Figure 12A), *Acidobacteria* showed an extremely significant positive correlation with SOM, STP, and Chl *a*, and an extremely significant negative correlation with NH_4_-N and DTN. *Cyanobacteria* showed an extremely significant positive correlation with COD_Mn_ and NH_4_-N and an extremely negative correlation with Chl *a*. *Chloroflexi* demonstrated an extremely negative correlation with WT, a negative correlation with DTN and NH_4_-N, a positive correlation with DO, and a significant correlation with Chl *a*. However, there were quite different correlations in sediment samples. In sediment, *Actinobacteria* was significantly positively correlated with CO and DO, while *Acidobacteriota* was extremely significantly negatively correlated with CO and DO and had a significant positive correlation with STP (Figure 12B). In addition, *Chloroflexi* demonstrated a negative correlation with DTN, and *Proteobacteria* was extremely significantly positively correlated with DTN, significantly positively correlated with NO_2_-N and DO, and significantly negatively correlated with SOM (Figure 12B).

## 4 Discussion

### 4.1 Diversity of bacterial communities in water and sediment

In the present study, bacterial DNA in water samples collected from all the sampling sites in July was not extracted successfully. This might be due to the water level fluctuation. Previous studies reported that the water quality of Poyang Lake was the best in summer (Wu et al., 2017), and the water level has a net positive effect on water quality through the dilution of environmental parameters, including the bacteria in the water (Ren et al., 2019). The water level at the time we sampled in July was 21.74 m (measured at Xingzi Station, an iconic hydrological station on Poyang Lake), which was at the historic high level of Poyang Lake (Liao and Kang, 2021). Because of the dilution, the amount of bacterial DNA in the 300 mL of water sample in July was not sufficient for the determination of bacterial diversity.

It is well known that *Proteobacteria* and *Actinobacteria* were most abundantly distributed in surface water and sediment in Poyang Lake (Dong et al., 2019; Ren et al., 2019; Zhao et al., 2020; Guo et al., 2023) and other lakes (Eiler et al., 2012; Mohiuddin et al., 2017). In the present study, the most abundant taxon in surface water during the extreme flood season was *Actinobacteriota* (33.45%), followed by *Cyanobacteria* (29.73%) and *Proteobacteria* (22.01%). While the composition of bacteria in sediment was more complex than in water, *Proteobacteria* (17.63%), *Actinobacteriota* (14.73%), *Chloroflexi* (14.63%), and *Acidobacteriota* (14.49%) occupied 60.78%. Previous studies highlighted that the different abundances of *Proteobacteria* and *Actinobacteria* in freshwater systems may be a result of different hydrological conditions, and the moderate variation in the properties of the water would not decrease their predominance (Warnecke et al., 2004; Zhao et al., 2020). According to our results, the extremely high water level (Xingzi Station: 21.74 m) did not decrease the predominance of these two groups in Poyang Lake. In the present study, the content of Cyanobacteria in water was much higher than that in sediment, which was 29.73% vs. 1.484% (*p* < 0.001). This was consistent with the finding of another research (Guo et al., 2023), which highlighted that Poyang Lake had a higher abundance of Cyanobacteria in water than that in sediment. In addition, our study also found that Cyanobacteria content was significantly different among the different sampling months (*p* < 0.001), Because of the significant correlations between Cyanobacteria biomass in water and the level of water eutrophication during the period of cyanobacterial blooms (Zhu et al., 2019), more attention must paid to the water quality of Poyang Lake (Qi et al., 2018; Zhang et al., 2021).

The distribution of TLI-PY in the whole Poyang Lake was relatively uniform (Qi, 2017), and the development of the bacterioplankton community composition in the euphotic layer showed a cyclical temporal pattern in freshwater ecosystems (Salmaso et al., 2017). In the present study, the bacterial diversity in the water of Poyang Lake during the extreme flood season showed clear temporal differentiation, while spatial differentiation was not pronounced. Such a phenomenon existed not only in these freshwaters but also in seawater (Meziti et al., 2015). In the present study, the bacterial community structure in the sediment showed obvious regionalism, and the sediment bacterial community structure from the Lake Bay and the channel area can be distinguished from each other, especially the phylum of *Proteobacteria*, *Acidobacteriota*, *Actinobacteriota*, *Nitrospirota*, and *Bacteroidota*. Similar results were reported in other lakes, which showed that the bacterial abundance and community structure of the lake sediment showed spatial heterogeneity in accordance with variations (Ding et al., 2015; Kou et al., 2016). In the present study, the Shannon index showed that there was no significant difference in bacterial diversity between the Sancha Bay and the Zhouxi Bay sediments, and this might be due to the geographical proximity of the Sancha Bay and the Zhouxi Bay areas, and the similarity of agricultural and fishing environments between these two lake bays (Zeng et al., 2023), and then the continuous heavy rain had caused a large amount of surrounding sediment to enter the lake.

In the present study, the difference in microbial diversity in sediment between the Sancha Bay and the channel was significantly higher than that between the Zhouxi Bay and the channel, and the microbial diversity gradually increased from the Sancha Bay to the Zhouxi Bay and then to the channel, but STN and STP concentrations showed opposite trends. This might be due to the anthropogenic disturbances from the extreme flood. Jin et al. (2019) reported that both physicochemical and microbiological parameters are indicated by anthropogenic disturbances in Poyang Lake, and N and P contents were not the main factors affecting bacteria abundance in the sediments of Poyang Lake (Guo et al., 2023). In addition, the exchange frequency of water flow (Adams et al., 2015) and depth (Zemskaya et al., 2020) have an impact on the bacterial structure in lakes.

Generally, the bacterial community and diversity in sediment are significantly higher than that in water. In the present study, the bacterial α-diversity indices of Poyang Lake were much lower in the water than that in the sediment. Similar results were found in Poyang Lake, and the habitat’s characteristics play an important role in microbiome formation (Sun et al., 2020; Guo et al., 2023).

### 4.2 Putative function profiles of microbial communities

In the present study, the most abundant groups in sediment and water were chemoheterotrophy, aerobic chemoheterotrophy, phototrophy, photoautotrophy, cyanobacteria, oxygenic photoautotrophy, animal parasites or symbionts, human pathogens pneumonia, hydrocarbon degradation, methylotrophy, chloroplasts, methanotrophy, fermentation, nitrogen fixation, and aromatic compound degradation. Compared to previous studies in Poyang Lake (Zhao et al., 2020), the abundance of genes related to human pathogens in our study is much higher. Zhao et al. (2020) reported that the major functions were chemoheterotrophy, phototrophy, photoautotrophy, cyanobacterial, oxygenic photoautotrophy, aerobic chemoheterotrophy, methylotrophy, and methanol oxidation, while functions such as animal parasites or symbionts, human pathogens all, hydrocarbon degradation, nitrification, and fermentation had a much lower presentation. However, the mean proportions of animal parasites or symbionts, human pathogens all, and human pathogen pneumonia in sediment were 9.66, 9.54, and 9.12%, respectively, and in water were 2.52, 2.41, and 1.78, respectively. This might be due to the flood water, which brought a high amount of pollutants (including pathogens) into Poyang Lake and posed a risk to human health. Since the possible origins of the water pathogens in the freshwater system include point sources (e.g., industrial wastewater and urban sewage) and non-point sources (e.g., land-based runoff containing wild and domestic animal excreta, leaking sewage, and agricultural effluent) (Pachepsky et al., 2011; Wan et al., 2022), large amounts of the above pollutants can enter lakes with surface runoff during heavy rainfall.

### 4.3 Microbial variation in the environment

Previous studies highlighted that the characteristics of BCC in water and sediment confirmed the significant correlations between bacterial diversity and levels of nutrients (Zhu et al., 2019; Guo et al., 2023). C, N, and P are recognized as key variables in microbial ecology due to their effects on the growth of certain bacteria in water and sediment (Zhang et al., 2020). In Poyang Lake, the nutrient concentrations in the water showed dramatic seasonal patterns (Ren et al., 2019), while the sediment nutrients showed obvious region and ecological niche distribution (Kou et al., 2016; Sun et al., 2020). This directly affected the bacterial community structure in both water and sediment. N, P, and other nutrients in the lake sediment are the endogenous load of the nutritive state of the lake. In the present study, the water bacterial diversity was not only correlated with the nutrient elements such as N and P in the water but also significantly correlated with the total P content in the sediment. This result might be due to the fact that the N, P, and other nutrients in the sediment will be released back into the water column under the mutual influence of various conditions (Cheng et al., 2020).

In the present study, during this extreme flood season, WT, NH_4_-N, SOM, Chl a, STP, and TP were significantly correlated with water microbial community structure, while SOM and STP were significantly correlated with sediment microbial community structure. That is to say that the bacterial community structure in the water column was not only sensitive to the geochemical characteristics of the water (WT, NH_4_-N, Chl *a*, and TP) but also affected by the nutrients in the sediment (SOM and STP). Lindström et al. (2005) and Lindström and Östman (2011) highlighted that temperature, pH, lake water retention time, and local conditions were closely related to BCC. In one terminal reservoir (Miyun Reservoir) of the South-to-North Water Diversion Project, the WT was positively correlated with the dominant bacteria; the total dissolved solids, TP, DO, and TN were the other factors that affected the structure and distribution of the water microbial community (Qu et al., 2018). In other water bodies, the relationship between microbial community diversity and WT was significantly correlated with each other (Chalifour et al., 2021). PH was also an important factor affecting bacterial diversity in many other habitats (Wang et al., 2016; Qian et al., 2018). However, in the present study, the pH values of water had little effect on the bacterial community. This might be due to the small change in the pH value (8.11 ± 0.36) in the water of Poyang Lake during the extreme flood season.

## 5 Conclusion

We revealed microbial structure and functions in Poyang Lake during the extreme flood season. Our study demonstrated that the bacterial community structure in water was greatly different from that in sediment in Poyang Lake during extreme flood seasons, and the dominant phyla in the water column were *Actinobacteriota, Cyanobacteria*, and *Proteobacteria,* while in sediment, the dominant phyla were *Proteobacteria*, *Actinobacteriota*, *Chloroflexi*, and *Acidobacteriota.* The bacterial community structure in the water column was affected not only by geochemical characteristics but also by the STP concentration in the sediment, and the bacterial diversity in the sediment was influenced by SOM and STP contents. In addition, the abundance of genes related to human pathogens in Poyang Lake was found, and the flood water with a high amount of pollutants is the main reason.

## Data availability statement

The original contributions presented in the study are included in the article/Supplementary material, further inquiries can be directed to the corresponding author/s.

## Author contributions

LZ: Conceptualization, Data curation, Formal analysis, Investigation, Methodology, Writing – original draft. LY: Formal analysis, Investigation, Writing – original draft. JX: Formal analysis, Writing – original draft. QL: Investigation, Writing – review & editing. DZ: Conceptualization, Data curation, Funding acquisition, Investigation, Supervision, Writing – original draft, Writing – review & editing. JL: Investigation, Writing – review & editing.

## Footnotes

^1^http://sourceforge.net/projects/rdp-classifier/

^2^http://www.arb-silva.de

^3^http://qiime.org/install/index.html
